# Medical Opinion and Movement

**Published:** 1910-04-30

**Authors:** 


### Medical Opinion and Movement.

### Treatment of Fractured Patella.

After suturing a fractured patella it is the usual practice to fix the knee in an extended position. Dr. Kausch, however, recommends that the knee be placed in a position of flexion to 100° to 110°. He commences massage immediately after operation, passive movements at the end of two or three days with increased flexion, and at the end of a fortnight he allows the patient to get up. Dr. E. W. Baum, of Kiel, has also adopted this method of flexion after operation, and has recently reported on the results obtained. He adopts a somewhat different method of treatment from Dr. Kausch. The knee is flexed only to 45° and is kept at rest for five or six days. Massage and passive movements are then commenced, and active movements at the end of a fortnight; but he thinks it advisable to delay getting up as long as possible, as it is difficult then to control the movements of the patient. A comparison of a series of six cases treated in this way with 14 cases treated with the knee extended shows results very favourable to the new method. The average duration of the treatment of the 14 cases with the knee extended was 64 days, and in only two cases the mobility of the knee was normal, or almost normal, at the time of leaving the hospital; whereas the average duration of the six cases treated with the knee flexed was 47 days, and the functional condition was so satisfactory in every case that no further treatment was required.

### Drainage of Douglas' Pouch.

For the drainage of purulent collections in Douglas's pouch after opening through the posterior wall of the vagina, Dr. Chaton, of Besançon, recommends a special circular drainage tube instead of the usual T-shaped tube. He points out that the T-shaped tube may not be to hand in the case of an urgent operation, and one made *ex tempore* is liable to be defective; on traction the transverse portion may separate from the longitudinal and its extraction require a further anæsthetic. It is not always easy to place in position, and its drainage capacity is not always satisfactory. Dr. Chaton's circular tube is readily made with an ordinary piece of rubber tubing, and appears to answer admirably to all the requirements of the case. The tubing should be eight to ten millimetres in diameter and about 50 centimetres long. The tube is bent on itself so as to form an elongated U, and the two arms are sutured together at the adjacent sides at a point from the bend so as to form a circle of about five centimetres diameter. The tube then assumes the form of a circle with two elongated ends. In the circular portion the usual holes are cut to assist drainage and this part is introduced into the cavity, while the parallel ends of the tube lie in the vagina and convey the secretion through the vagina to the vulva. By seizing the circular portion laterally in forceps it is easily introduced into the cavity, and on releasing it from the forceps it assumes its circular shape and remains in position. The cavity can then be thoroughly irrigated by syringing alternately through the two ends of the tube. The tube is easily withdrawn by simple traction.

### Tetanus after Injections of Quinine.

Dr. Rigollet, in *La Presse Médicale*, discusses the question of tetanus following hypodermic injections of quinine. In his opinion such a disaster is due to contamination, and not to any inherent fault of the drug. As Vincent has shown, if tetanus bacilli be injected soon after a hypodermic injection of an insoluble quinine salt has been given, the organisms will be found in the greatest abundance, not at the site of their own injection, but at that of the quinine; and probably this fact is due to the influence of the drug in paralysing leucocytosis. The authors who reported cases of tetanus following quinine injection claim to have used the most rigid anti- and aseptic precautions, and attribute the disease to latent spores in the patient's organism, which developed as the result of the paralysing action of the drug. Owing to the general use of hypodermic injections in quartan fever, it is important that some definite decision should be come to with regard to this method of administration. Rigollet, in attempting this, has investigated the original communications, and finds that in nearly every case abscesses were formed at the site of injection, through which the organisms of suppuration and of tetanus could have gained admission. He therefore thinks that in all cases faulty technique was the cause of the disaster, an opinion which is strengthened by the fact that no case has been reported for several years in spite of the thousands of injections which are practised annually. As, however, there is always a danger of an accident happening, he is of opinion that injections should only be given by a physician and with every precaution; the neutral chloride should be used, as it destroys tetanus spores, and the fluid should be injected into the muscles and not subcutaneously.

### Locomotor Ataxia and Radioscopy.

In a contribution to *Le Progrès Médical* Dr. A. Varet, of the electrotherapeutic department of La Charité Hospital, describes a new clinical sign which can be elicited by means of radioscopy, and which should prove useful in the diagnosis of locomotor ataxia in its early stage. If a normal individual be instructed to breathe slowly and deeply and the incursions of his diaphragm be watched on the fluorescent screen it will be found that both sides of the diaphragm are raised and depressed synchronously and in perfect rhythm. Moreover, the incursions of the diaphragm are regular, so that the movements of successive inspirations and expirations can be superimposed. In a tabetic patient, however, the diaphragm displays the same incoordination and ataxia as the other skeletal muscles of the body, and, according to this author, often in a marked degree at an early stage of the disease. The incursions of the diaphragm are irregular in rhythm, the two sides do not move synchronously, and the successive movements are unequal both in point of duration and amplitude. There is, in fact, the same irregularity and lack of control displayed in the respiratory movements of the diaphragm which characterise the ataxia of the limbs of the tabetic subject. This irregularity in the movements is accompanied by an exaggerated depression of the dome of the muscle owing to a loss of muscular tone. The author, therefore, gives four characteristics to the tabetic diaphragm as revealed by radioscopy—arrhythmia, asynergia, inco-ordination, and hypotonia. He points out further that radioscopic examination of patients may not infrequently reveal an early stage of ataxia previously unsuspected. The introduction of the patient into the darkened room may disclose the lack of muscular control when deprived of the visual sense which has not been before in evidence unless Romberg's sign has been specially sought for.

### Sympathetic Ophthalmia.

In the *Ophthalmoscope* Dr. Gifford, of Nebraska, gives particulars of the salicylate treatment of sympathetic ophthalmia, which he has been advocating in America for the past eleven years. The principle on which he acts is to give the patient one grain of salicylate of soda *per diem* for every pound of body weight. If this heroic dosage produces no good effect, he is even prepared to increase it thirty per cent. Unless there is personal idiosyncrasy, he says, this amount can be tolerated for four to seven days, provided the patient lies quietly in bed. About thirty grains is given at a time with two drachms of brandy in half a tumbler of water. When active signs of inflammation have disappeared, he continues the treatment on two days out of three for two or three weeks longer. The only ill-effect is occasional delirium, but even this has never in the author's experience done any harm; he orders the treatment not only for sympathetic ophthalmia, but also for iritis, interstitial keratitis, and traumatic infections. He does not neglect other treatment, but rather the reverse; the salicylate is given in addition to mercurial inunction and any local measures, such as the use of atropine, that may be necessary. Since 1896 he has employed the salicylate method in 16 cases of this disease; the results have been in two cases bad, in one moderate, in one good, and in twelve very good. This is a record which is certainly far in advance of anything hitherto in vogue in this country for this very dangerous and terrible malady.

### Luxation of the Eyeball.

A curious accidental and unintentional dislocation of the eyeball produced in the extraction of a foreign body from the cornea suggests to Mr. A. R. Galloway the novel idea that such luxation, intentionally caused, might prove a most valuable aid to diagnosis and treatment. The patient was a young man; after instilling cocaine the surgeon was using his first and second fingers in the usual way to retract the lids and steady the globe, when the eye suddenly shot quite out of the orbit on to the cheek, the lids closing behind it. The patient suffered pain, and complained of feeling sick; he was, and remained, quite in ignorance of what had really happened. There was no great difficulty in replacing the eye by firm pressure, and the foreign body was then successfully removed. The vision in that eye, tested soon after, was found to be $\frac{6}{18}$; three days later it was $\frac{6}{6}$, and there was no abnormality of disc or fundus. After this unusual experience Mr. Galloway attempted luxation of many globes when dealing with foreign bodies. Stopping short of actual luxation, he satisfied himself that in many cases he could have produced it by the slightest extra tension on the lids; and he says he shall not hesitate to try this novel aid to diagnosis in any case in which it seems justifiable. Of such cases he gives two instances, from his own past experience, of deep, penetrating wounds. Where counter-puncture at the back of the eye is doubtful, information thus gained may prevent an unnecessary enucleation.

### The Incidence of Adenoids.

As the result of the very extensive experience gained among London elementary school children in the capacity of school medical officer under the County Council, Mr. Macleod Yearsley sums up in the *British Journal of Children's Diseases* his conclusions from the statistics he has compiled. He finds that 37 per cent. of the children have adenoids, and that 72 to 76 per cent. of these have enlarged tonsils also. About a third of adenoid cases are mouth breathers; but this result can be brought about by tonsils alone without appreciable adenoids. Adenoids are commonest at about eight years old, and again at twelve; the incidence upon the two sexes is equal. The so-called adenoid facies is uncommon except in association with a marked degree of adenoid growths. The abnormally high palate is more probably a congenital cranial peculiarity than a result of acquired nasal stenosis, and if there is any causal connection at all it may be that the high narrow palate disposes to adenoids rather than *vice versâ*. The presence of adenoids has more to do with that of carious teeth than have mouth breathing and palate shape, probably due to the increased tendency to oral sepsis in adenoid children. Irregularity of the upper incisors, on the other hand, is not due to adenoids so much as to palate shape. Some eleven per cent. of adenoid cases develop ear complication, and adenoids are probably by far the most important factor in the ætiology of aural disease in childhood.

### Gastro-Enterostomy with Clamps.

Several surgeons have contributed to a discussion in the *Lancet* on the occurrence of hæmorrhage after gastro-enterostomy with clamps. Mr. Sinclair White reported a case first of all early this year, and following that several other operators came forward with similar experiences. Finally, Dr. Ollerenshaw, a house surgeon at the Manchester Infirmary, was able to report two cases; and Mr. Platt, who was the operator on each occasion, concludes with a suggestion as to the technique of gastro-enterostomy with a view to avoiding such disquieting accidents for the future. In both the Manchester cases the patients went on normally for twenty-four hours or more, and then displayed suddenly and spontaneously the symptoms of severe internal hæmorrhage. Both recovered, and both bore out the diagnosis by the evacuation of large tarry stools a few hours after the sudden collapse. Mr. Platt thinks there is little risk of hæmorrhage from the duodenum, and that it is the stomach which bleeds. He suggests that after the posterior rows of sutures are inserted the stomach clamp should be loosened and the main bleeding points picked up. The clamp can then be reapplied and the anterior suturing completed. The advantages of clamps in this operation are so great that they are not likely to be discarded, even though it were certain that occasional hæmorrhages followed their use; and therefore this plan of loosening the clamps before tightening the suture line seems well worth a trial by operating surgeons.

### The Arsenic Ion in Skin Disease.

In *Les Annales d'électricité et de radiologie*, Dr. Winckler discusses the treatment of certain skin affections by ionisation with arsenic. He has found this form of medication particularly useful in dealing with psoriasis, and in a case of disseminated sarcomatosis of the skin. In this case the internal administration of arsenic was inefficacious. He finds that after ionisation the local action is confined to the points treated. There is no general reaction, nor are there any other signs of arsenical poisoning. In psoriasis the crusts rapidly fall off, hyperæmia disappears, and the condition is cured without leaving behind the usual pigmented areas; in fact, as the result of treatment by ionisation patches of pigment tend to disappear. The author usually uses a solution of arsenious acid of a strength of 1 in 200; the treatment is repeated every second day for 10 minutes, and the drug introduced at the kathode. Arsenic acts as a vaso-constrictor of the skin vessels, and part at least of its curative effects are due to this action.

### Treatment of Surgical Shock.

Dr. J. H. Hopkins, in the *Boston Medical and Surgical Journal*, describes a method of treating post-operative abdominal shock, which he considers superior to that used ordinarily. He attempts to overcome the shock by administering a shock of another kind to the vaso-motor mechanism, with the application of hot saline solution at 112° F. to the nerves of the splanchnic area. To this end two of the abdominal sutures nearest the umbilicus are removed and through the opening thus made a glass tube is inserted under the omentum and transverse meso-colon, so as to reach the posterior peritoneum in the neighbourhood of the solar plexus and sympathetic ganglia. The glass tube is then connected up with a reservoir containing hot saline solution, and a pint of the fluid allowed to flow. The immediate result is a marked rise in blood-pressure owing to the vascular contraction which follows the irritation by the fluid of the splanchnic and sympathetic nerves. The abdominal wound is then covered with gauze and adhesive plaster, and pressure sustained by an abdominal binder. This treatment is followed by two-hourly rectal injections of half a pint of hot saline, the patient otherwise being kept absolutely quiet.

### Mercury in the Treatment of Syphilis.

A serious indictment of the indiscriminate use of mercury, and especially of the "intensive" treatment when dealing with a case of syphilis, has recently appeared in *Le Journal des Praticiens* from the pens of Huchard and Fiessinger. The authors point out that the most divergent opinions are held as to dosage in any given case, and that the actual dose given depends more on the state of mind of the practitioner than on the state of the patient. Mercury is useless as a prophylactic in syphilis, as may be seen from the fact that workers in the mines of Almaden, who are in a chronic state of saturation with the drug, contract the disease in the ordinary way. Again, the drug does not prevent the appearance of para-syphilitic nervous affections, and, moreover, when given in the form of an ointment, it acts equally well on tuberculous and other chronic suppurations as on syphilitic lesions. Systematic mercurialisation in the silent period of syphilis does not seem to have any obvious advantages, for if the drug does not meet with the specific organism, it exhausts its energies on the cellular tissues, and especially on those of the nervous and renal systems. Since the general adoption of the intensive method of treatment, signs of arterial hypertension are more frequently met with, and interstitial nephritis and cardiac disturbances are not slow in making their appearance. It is not always understood that a patient whose syphilitic condition has improved is much more sensitive to the action of mercury than another who is suffering from a recent attack of the disease, and a consequence of this misunderstanding is, that a patient who is in a condition of neurasthenia and subject to nervous derangement as the result of intensive treatment, is given further mercurial treatment to cure this condition! Finger's axiom must therefore be remembered: that the most energetic mercurial treatment is not capable of preventing relapses even in benign cases. Heavy doses should therefore not be given at the start, and no treatment should be commenced without an examination of the urine and of the arterial tension. The authors believe that healthy individuals are best treated with pills, such as the sub-iodide of mercury pill, in daily doses of 1 to 1½ grain for six weeks, followed by a period of rest. Local treatment suffices for the chancre. Injections are useful to patients whose digestion is poor, and are best given with soluble salts in the case of private patients. Insoluble solutions are difficult to control, and should be reserved for patients of the hospital class.

